# Serum levels of soluble Fas, soluble tumor necrosis factor-receptor II, interleukin-2 receptor and interleukin-8 as early predictors of hepatocellular carcinoma in Egyptian patients with hepatitis C virus genotype-4

**DOI:** 10.1186/1476-5926-9-1

**Published:** 2010-01-05

**Authors:** Abdel-Rahman N Zekri, Hanaa M Alam El-Din, Abeer A Bahnassy, Naglaa A Zayed, Waleed S Mohamed, Suzan H El-Masry, Sayed K Gouda, Gamal Esmat

**Affiliations:** 1 Virology and Immunology Unit, Cancer Biology, National Cancer Institute, Cairo University, Cairo, Egypt; 2 Pathology Department, National Cancer Institute, Cairo University, Cairo, Egypt; 3 Tropical Medicine Department, Faculty of Medicine, Cairo University, Cairo, Egypt; 4 Biochemistry Department, Faculty of Science, Cairo University, Giza, Egypt

## Abstract

**Background:**

Liver disease progression from chronic hepatitis C virus (HCV) infection to hepatocellular carcinoma (HCC) is associated with an imbalance between T-helper 1 and T-helper 2 cytokines. Evaluation of cytokines as possible candidate biomarkers for prediction of HCC was performed using soluble Fas (sFas), soluble tumor necrosis factor receptor-II (sTNFR-II), interleukin-2 receptor (IL-2R) and interleukin-8 (IL-8).

**Results:**

The following patients were recruited: 79 with HCV infection, 30 with HCC, 32 with chronic liver disease associated with elevated liver enzyme levels (with or without cirrhosis) in addition to 17 with chronic HCV with persistent normal alanine aminotransferase levels (PNALT). Nine normal persons negative either for HCV or for hepatitis B virus were included as a control group. All persons were tested for sFas, sTNFR-II, IL-2R and IL-8 in their serum by quantitative ELISA. HCC patients had higher levels of liver enzymes but lower log-HCV titer when compared to the other groups. HCC patients had also significantly higher levels of sFas, sTNFR-II and IL-2R and significantly lower levels of IL-8 when compared to the other groups. Exclusion of HCC among patients having PNALT could be predicted with 90% sensitivity and 70.6% specificity when sTNFR-II is ≥ 389 pg/ml or IL-8 is < 290 pg/ml.

**Conclusions:**

Serum TNFR-II, IL-2Rα and IL-8, may be used as combined markers in HCV-infected cases for patients at high risk of developing HCC; further studies, however, are mandatory to check these findings before their application at the population level.

## Background

Hepatocellular carcinoma (HCC) ranks as the fifth most common cancer around the world and the third most frequent cause of cancer-related death. It represents the most common primary malignant tumor of the liver and is one of the major causes of death among patients with cirrhosis [1]. The increased incidence of HCC in the United States as well as in Japan over the past 20 to 30 years [2,3] has been partially attributed to the emergence of the hepatitis C virus (HCV), an established risk factor for developing HCC [4,5]. The prevalence of HCV infection varies significantly; higher rates have been reported in African and Asian countries, whereas industrialized nations in North America, northern and western Europe, and Australia had lower prevalence rates [6]. Egypt has the highest prevalence of HCV in the world, ranging from 6 to 28% [7-10], with an average of approximately 13.8% in the general population and there is an expected increase in hepatitis C-related mortality in that country [11].

The continued viral replication and persistent attempt by a less than optimal immune response to eliminate HCV-infected cells are implicated in hepatocyte aberrations, accumulation of chromosomal damage and possibly initiation of hepatic carcinogenesis [12]. The prognosis of HCC is generally most serious with a great need for serum markers that could be used for its early detection and, consequently, to start a therapeutical procedure as soon as possible, potentially at a curable phase. Serum α-fetoprotein (AFP) levels are frequently not elevated at a significant proportion in patients with early-stage, potentially curable, HCC. Therefore, other markers should have been studied in an attempt to identify a more sensitive laboratory test.

Cytokines are small secreted proteins which regulate immunity, inflammation and haematopoiesis in connection with liver disease progression due to chronic HCV infection, which is associated with an imbalance between pro- and anti-inflammatory cytokines. Therefore, elevated serum cytokines could be a risk factor for the occurrence of HCC in patients with HCV related chronic hepatitis and cirrhosis. Cytokines were shown to be used as biomarkers for early detection of HCC [13] in addition to their possible use as potential predictors for interferon (IFN) treatment in HCV genotype-4 patients [14]. Several cytokines are involved in the process of HCC invasion and metastasis, including soluble Fas (sFas), soluble tumor necrosis factor receptor-II (sTNFR-II), interleukin-2 receptor (IL-2R) and interleukin-8 (IL-8). As the knowledge of tumor biology becomes progressively clear, more and more new biomarkers with high sensitivity and specificity could be found and then routinely used for clinical assays.

The sFas, obviously increased in HCC with a significant difference between patients of chronic liver disease (CLD) and normal controls, was found to correlate with the severity of liver disease and to resist the occurrence of HCC apoptosis [15,16]. In chronic hepatitis B virus (HBV) or HCV infected patients, serum IL-2R was used both to screen high-risk patients and to monitor treatment responses in patients with hepatitis who develop HCC. Serum IL-2R appeared not only with a significantly greater frequency than AFP, but was a more sensitive marker of successful treatment and recurrence of HCC as well [17].

Circulating TNF-α level increases during HBV [18-22] and HCV infection [18,23-26] and is correlated with the severity of hepatic inflammation, fibrosis and tissue injury [18,22,24,27]. TNF-α plays a role in initiating fibrogenesis through binding to specific cellular receptors; *i.e*., TNFRs [28], which can be proteolytically cleaved into two soluble forms: sTNFR-I and sTNFR-II. High concentration of sTNFR-II has been observed for prolonged periods in the circulation of patients with various inflammatory diseases (including HCV infection), making sTNFR-II an ideal serum biomarker for characterizing type 1 immune response [29-32]. Moreover, IL-8 contributes to human cancer progression through potential mitogenic, and angiogenic functions. IL-8 expressions plays a more critical role in the metastatic potential of human HCC (such as vascular invasion) than in angiogenesis or tumor proliferation [33]. Our aim was to evaluate the serum levels of sFas, TNFR-II, IL-2R and IL-8 as possible candidate biomarkers for an early detection of HCC.

## Results

**Figure 1:**
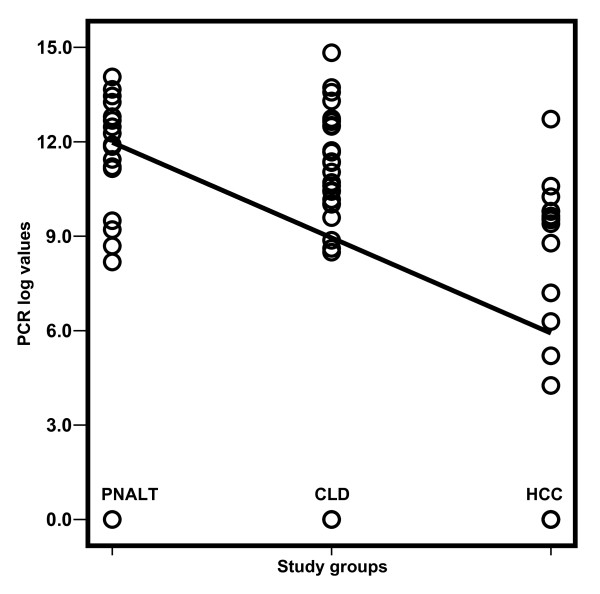
**Scatter diagram of the distribution of log-HCV titer results among the different study groups**. PNALT: Chronic hepatitis C with persistent normal alanine aminotrasferase; CLD: Chronic liver disease; HCC: hepatocellular carcinoma.

The clinical characteristics of the studied groups are shown in Table 1. All recruited patients were positive for HCV antibodies, PCR for HCV RNA and all had genotype-4. Mean age of patients with HCC was significantly higher than that of the other groups (*p *< 0.001). Liver function tests were significantly elevated, whereas log-HCV titer was significantly lower in HCC patients (*p *< 0.001) when compared to patients with chronic hepatitis C with persistent normal alanine aminotransferase levels (PNALT) and chronic liver disease (CLD) patients. Figure 1 shows the distribution of log-HCV titer in the different study groups, which included 68 men and 29 women. Mann-Whitney test was used for comparing log-HCV, sFas, sTNFR-II, sIL-2R and IL-8 values with gender. Comparing the means of men *versus *women, the former had only higher and significant (*p *= 0.04) log-HCV titer (11.16 ± 4.1) and (9.7 ± 1.5), respectively; however, all other markers did not statistically differ.

**Table 1:** Patients characteristics and log-HCV titer among the different study groups

Variables	Control (9)	PNALT (17)	CLD (32)	HCC (30)	*p*-value
M/W	7/2	12/5	24/8	25/5	< 0.001

Age (years): Mean ± SD	50.9 ± 4.6^b^	35.1 ± 11.5^c^	43.4 ± 8.7^b^	60.7 ± 8.3^a^	< 0.001

Log HCV-titer	<615*	10.9 ± 3.2^a^	9.9 ± 4.1^a^	5.2 ± 4.7^b^	< 0.001

Groups with similar letters are not different statistically. A *p*-value < 0.05 was considered significant. M/W: Men/Women; PNALT: chronic hepatitis C with persistent normal alanine aminotrasferase; CLD: chronic liver disease; HCC: hepatocellular carcinoma. *All cases were under detection limit (<615 IU/ml) and so they were not included in the statistical analysis (Kruskal-Wallis ANOVA).

**Figure 2:**
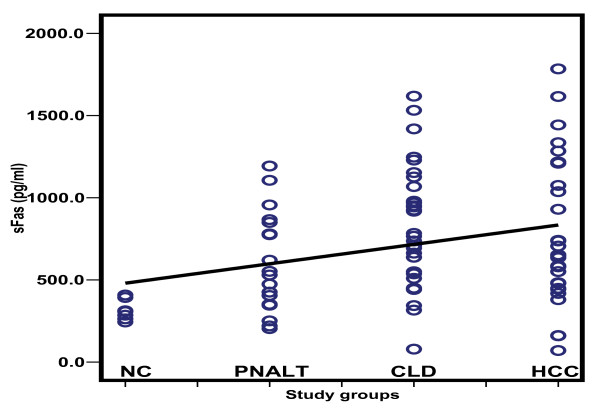
**Scatter diagram representing the distribution values of sFas in the different study groups**. NC: normal controls; PNALT: Chronic hepatitis C with persistent normal alanine aminotrasferase; CLD: Chronic liver disease; HCC: hepatocellular carcinoma.

**Figure 3:**
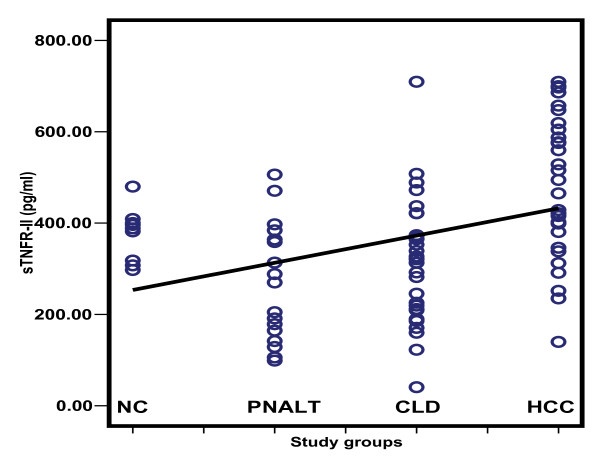
**Scatter diagram representing the distribution values of sTNFR-II in the different study groups**. NC: normal controls; PNALT: Chronic hepatitis C with persistent normal alanine aminotrasferase; CLD: Chronic liver disease; HCC: hepatocellular carcinoma.

**Figure 4:**
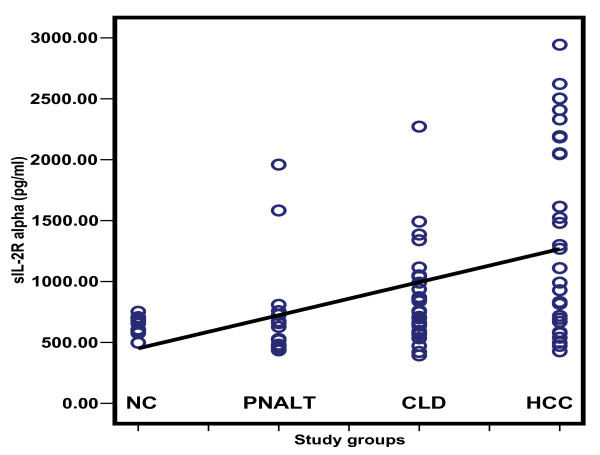
**Scatter diagram representing the distribution values of sIL-2Rα in the different study groups**. NC: normal controls; PNALT: Chronic hepatitis C with persistent normal alanine aminotrasferase; CLD: Chronic liver disease; HCC: hepatocellular carcinoma.

**Figure 5:**
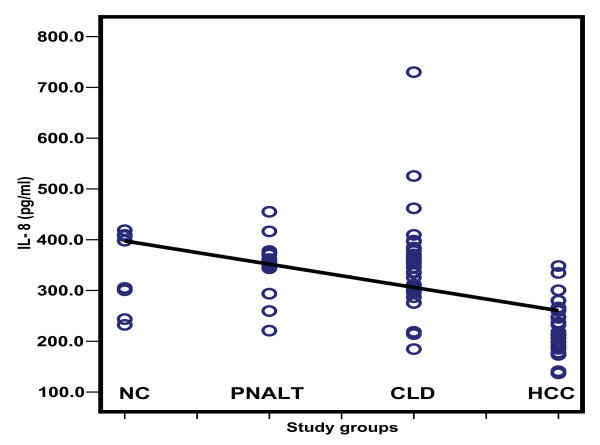
**Scatter diagram representing the distribution values of IL-8 in the different study groups**. NC: normal controls; PNALT: Chronic hepatitis C with persistent normal alanine aminotrasferase; CLD: Chronic liver disease; HCC: hepatocellular carcinoma.

Table 2 depicts the comparison of the serum levels of sFas, sTNFR-II, sIL-2Rα and IL-8. HCC patients had higher sFas, sTNFR-II and sIL-2R than patients with PNALT, CLD and normal controls with a significant difference for sFas between HCC patients and control (*p *< 0.001). The sTNFR-II was significantly elevated in HCC patients compared to those with PNALT and CLD (*p *< 0.001), whereas sIL-2R was significantly elevated in HCC patients when compared to those with PNALT patients and control. On the other hand, IL-8 was significantly lower among HCC patients when compared to the other groups (*p *< 0.001); but with no significance between the other groups. The scatter diagrams of the studied cytokines in the different study groups are shown in Figures 2, 3, 4 and 5.

**Table 2:** Serum levels of sFas, sTNFR-II, sIL-2R and IL-8 in the different study groups.

Cytokines (pg/ml)	Control	PNALT	CLD	HCC	*p*-value
sFas	316 ± 62.5^b^	605.82 ± 304^ab^	814.94 ± 362^a^	762.18 ± 437^a^	< 0.001

sTNF-RII	375.26 ± 58.4^ab^	268.58 ± 129^b^	315.27 ± 133.5^b^	480.16 ± 154.4^a^	< 0.001

sIL-2Rα	639.84 ± 78.7^b^	710.10 ± 422^b^	845.38 ± 385.2^ab^	1372.58 ± 779.6^a^	0.001

IL-8	345.84 ± 75.6^a^	350.7 ± 53.6^a^	352.33 ± 98.3^a^	228.61 ± 51.1^b^	< 0.001

Values are expressed as mean ± SD. Groups with similar letters are not statistically different. A *p*-value < 0.05 was considered significant; PNALT: chronic hepatitis C with persistent normal alanine aminotrasferase; CLD: chronic liver disease; HCC: hepatocellular carcinoma.

Correlation was done between the serum levels of the studied cytokines, liver enzymes and log-HCV titer. The liver enzymes, aspartate aminotransaminase (AST), alanine aminotransferase (ALT), and alkaline phosphatase, were significantly correlated with sTNFR-II, sIL-2R and IL-8, as exhibited in Table 3.

**Table 3:** Correlation of different markers, liver enzymes showing Pearson's r value and *p*-values

Labs	ALT	ALP	log-HCV titer	sFas	sTNFR-II	IL-2R	IL-8
AST	0.55 (0.000)	0.497 (0.000)	-0.481 (0.000)	0.127 (0.3)	0.265 (0.029)	0.332 (0.006)	-0.415 (0.000)

ALT		0.590 (0.000)	0.027 (0.828)	0.338 (0.002)	0.253 (0.021)	0.392 (0.000)	-0.269 (0.014)

ALP			-0.218 (0.083)	0.081 (0.5)	0.342 (0.004)	0.374 (0.002)	-0.488 (0.000)

log-HCV titer				0.006 (0.96)	-0.220 (0.067)	-0.170 (0.15)	0.488 (0.000)

sFas					0.276 (0.010)	0.403 (0.000)	-0.139 (0.199)

sTNFR-II						0.598 (0.000)	-0.304 (0.004)

IL-2R							-0.236 (0.028)

Correlation is significant at the level of α < 0.05. The *p*-value appears within brackets. AST - aspartate aminotransaminase; ALT - alanine aminotransferase; ALP - alkaline phosphatase.

A statistically significant correlation was found between log-HCV RNA, sTNFR-II and IL-8 (*p *= 0.06 and 0.000) respectively, whereas sIL-2R and sFas did not show any significant difference in relation to log-HCV titer.

Moreover, correlation studies revealed a significant correlation between sFas, in the one hand, and sTNFR-II or IL-2R, in the other hand (*p *= 0.01 and 0.000, respectively); but not with IL-8. The sTNFR-II was significantly correlated with sFas, IL-2R or IL-8 (p = 0.01, 0.000 and 0.004, respectively). IL-2R was significantly correlated with either sFas or IL-8 (*p *= 0.000 and 0.02, respectively). IL-8 was negatively correlated with sTNFR-II or IL-2R (*p *= 0.000 and 0.02, respectively).

In the present study, levels of AFP among HCC patients were ≥ 200 ng/ml in 9 patients, whereas 11 patients had levels < 200 ng/ml. There was no statistically significant difference when the levels of AFP were assessed against the serum levels of any of the studied cytokines.

**Figure 6:**
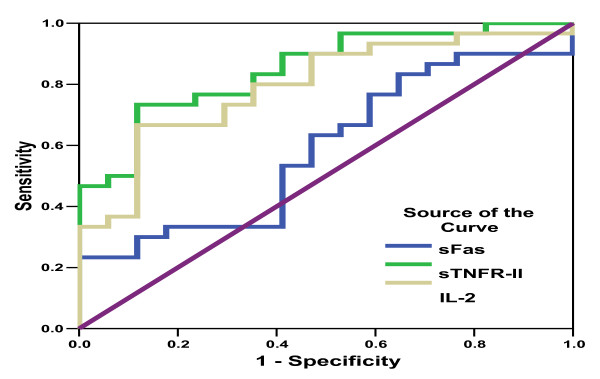
**ROC (Receiving operating characteristic) curve showing sFas, sTNFR-II and IL-2Rα in PNALT**. Chronic hepatitis C with persistent normal alanine aminotrasferase) *versus *HCC (hepatocellular carcinoma) patients.

**Figure 7:**
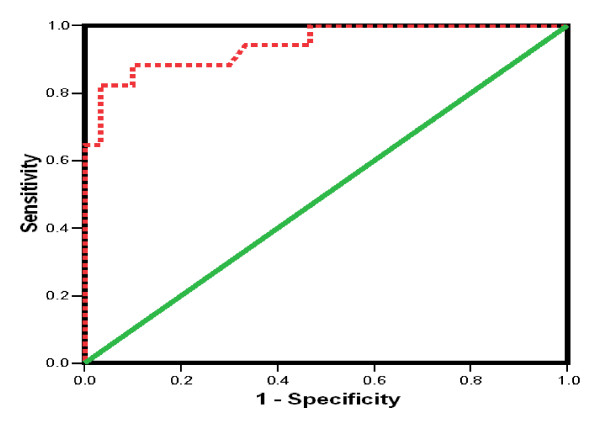
**ROC (Receiving operating characteristic) curve showing IL-8 in PNALT (chronic hepatitis C with persistent normal alanine aminotrasferase) *versus *HCC (hepatocellular carcinoma) patients**.

Receiving operating characteristic (ROC) analysis curves and the corresponding area under the curve were calculated for providing the accuracy of the cytokines in differentiating between the different groups under consideration. Sensitivity (*i.e*., true positive rate), specificity (*i.e*., true negative rate), positive predictive value, negative predictive value and cutoff values showing the best equilibrium between sensitivity and specificity were evaluated. ROC curve and best cutoff values were calculated for patients with PNALT and HCC because there was no good discrimination between the other groups. ROC curve values for sTNFR-II and IL-8 among PNALT and HCC patients yielded a cutoff of 398 pg/ml and 345 pg/ml, respectively, as shown in Table 4, and Figures 6 and 7. ROC curve for IL-2R and sFas is shown in Figure 6.

**Table 4:** ROC curve values for sTNFR-II and IL-8 in PNALT and HCC patients

ROC values	sTNF-RII ≥ 398	IL-8 ≥ 345	TNFR-II ≥ 398 or IL-8 <290
Sensitivity	73.3%	96.7%	100%

Specificity	88.2%	76.5%	70.6%

AUC	0.849	0.588	0.794

NPV	65.2%	92.2%	100%

PPV	91.7%	87.9%	85.7%

ROC - receiving operating characteristic; AUC - area under the curve; NPV - negative predictive value; PPV - positive predictive value; PNALT: Chronic hepatitis C with persistent normal alanine aminotrasferase. HCC: hepatocellular carcinoma.

Further analyses on the cytokines in HCC and PNALT patients are shown in Table 5. Only sTNFR-II and IL-8 levels among patients with PNALT and HCC were analyzed. There were no satisfactory cutoff values for either IL-2R or sFas for both specificity and sensitivity, *i.e*., one on the expense of the other as evident by the ROC curve.

**Table 5:** sTNFR-II and IL-8 levels in PNALT and HCC cases

Cytokines (pg/ml)	PNALT, N = 17	HCC, N = 30	*p*-value
sTNFR-II ≥ 398	2 (11.8%)	22 (73%)	0.000

sTNFR-II < 398	15 (88.2%)	6 (27%)	0.000

IL-8 < 345	4 (23.5%)	29 (97%)	0.000

IL-8 ≥ 345	13 (76.5%)	1 (3.3%)	0.000

TNFR-II ≥ 398 or IL-8 <290. Either + ve	5 (29.4%)	30 (100%)	0.000

TNFR-II ≥ 398 and IL-8 <290. Both - ve	12 (70.6%)	0 (0%)	0.000

TNFR-II ≥ 398 and IL-8 <290. Both + ve	0 (0%)	21 (70%)	0.000

Others	17 (100%)	9 (30%)	0.000

PNALT: chronic hepatitis C with persistent normal alanine aminotrasferase; HCC: hepatocellular carcinoma.

Among the HCC patients, 22/30 (73.3%) had mean sTNFR-II levels of ≥ 398 pg/ml, whereas only 2/17 (11.8%) cases with PNALT had this value with a highly significant difference (*p *= 0.000). Regarding IL-8, 29/30 (96.7%) HCC patients had IL-8 level < 345 pg/ml compared to only 4/17 cases with PNALT, whereas most PNALT patients had IL-8 ≥ 345 pg/ml (*p *= 0.000). When both sTNFR-II and IL-8 were combined together, all HCC cases 100% had either sTNFR-II ≥ 398 pg/ml or IL-8 < 290 pg/ml (*p *= 0.000) and 21/30 (70%) HCC had sTNFR-II ≥ 398 pg/ml and IL-8 < 290 pg/ml compared to none of PNALT cases (*p *= 0.000). In this vein, combined assessment of both sTNFR-II and IL-8 at a cutoff of ≥ 398 pg/ml and < 290 pg/ml, respectively, would be better in the diagnosis of HCC than either of them individually.

## Discussion

HCC generally develops following an orderly progression from cirrhosis to dysplastic nodules to early cancer development, which can be reliably cured if discovered before the development of vascular invasion [34]. Early detection of HCC in those patients provides the best chance for a curative treatment, but AFP levels are frequently normal in patients with small HCC and are not elevated in a significant proportion of patients with early-stage, potentially curable HCC.

Elevated concentrations of cytokines represent a characteristic feature of CLD, regardless of the underlying etiology, and may represent a consequence of liver dysfunction instead of an inflammatory disorder [35]. Cytokines imbalance between T-helper 1 (Th1) and T-helper 2 (Th2) can prolong inflammation, leading to necrosis, fibrosis and CLD [36] in addition to the development and progression of HCC [37]. Cytokine production is thought to play an important role in the recruitment of tumor associated inflammatory cells, induction of angiogenesis and direct modulation of tumor cell proliferation [38,39]. The cytokines studied in this work were carefully chosen to include cytokines of the Th1 repertoire (IL-2R and sTNFR-II), in addition to one of the important pro-inflammatory cytokines (IL-8), and other factors as sFas.

In the present study, liver function tests were significantly elevated whereas log-HCV titer was significantly lower in HCC patients (*p *< 0.001) when compared to PNALT and CLD patients. In agreement with our findings, HCC group had the highest values (86.3%) for various concurrently-measured liver function tests, significant higher values of AST/ALT, ALT, AST (each, *p *< 0.001) than cirrhotic patients as previously reported [40]. On the other hand, HCV levels were markedly higher in non-cancerous liver than in HCC (*p *= 0.001) [41]. Moreover, comparing HCV titers of four HCC isolates and surrounding cirrhotic liver tissues in two anti-HCV positive patients; the copy numbers of HCV-RNA were 1 × 10^6 ^and 4 × 10^6^/gm wet weight of HCC, and 8 × 10^7 ^and 3.2 × 10^8^/gm wet weight of cirrhotic liver tissues from patient-1 and -2, respectively [42]. The present study showed that men had higher log-HCV RNA titer than that detected in women; then, a strong evidence is provided in favour of a higher HCV clearance rate in women compared with that in men [43].

Fas (APO-1 or CD95) is a cell-surface receptor that transduces apoptotic signals from Fas ligand (Fas-L) [44]. Apoptosis is tightly regulated throughout a variety of mechanisms, one of which is postulated to be the production of soluble forms of Fas (sFas) that normally binds to Fas-L, thus blocking the signaling of the membrane-bound form of Fas. Peripheral blood mononuclear cells in HCV infection exhibit decreased susceptibility to Fas-L induced cell death. This may signify a mean by which HCV escapes immune surveillance; however, it would be worth a further investigation on this phenomenon. The sFas appeared to increase in advanced stages of HCV-induced liver disease, as a result of host-related immunological factors [45]. In the present series, the mean values of sFas were significantly higher in HCC patients compared to the other groups (*p *< 0.001). This could be explained by the role of sFas in the inhibition of apoptosis, progression to end stage liver damage, and subsequent development of HCC. Similarly, a significant elevation of serum levels of sFas in HCC patients compared with liver cirrhosis and healthy control was previously reported [46]. Previous studies [47,48] have reported mRNA encoding secreted sFas in a number of hepatitis and HCC cases indicating that sFas may function as an inhibitor of the Fas/Fas-L system and escape of tumor cells from immune surveillance may then occur. In chronic hepatitis, sFas was correlated with the severity of disease [15] and its expression can illustrate the mechanism of liver injury caused by death receptors throughout the multistep process of fibrosis/carcinogenesis. So, the increased incidence of HCC is correlated not only with the higher degree of hepatic fibrosis, but also with the lower expression of Fas protein [49].

The rate of progression to end-stage liver disease might be related to an up-regulation of the TNF-α/Fas pathways and an age-dependent host response [50]. Pro-inflammatory TNF-α released by host and tumor cells is an important factor involved in initiation, proliferation, angiogenesis as well as metastasis of various cancer types [51]. Activities of TNF-α are mediated through TNFR-I and TNFR-II [52]. Our results showed that levels of sTNFR-II were elevated in patients with PNALT, CLD and HCC with a significant difference between HCC in relation to the other two groups (*p *< 0.001). These results are in agreement with previous published results [13,29,53], where it was found that sTNFR-IIα were closely correlated with disease progression in chronic HCV infection. Enhanced TNF-α and TNFRs in chronic HCV infection may reflect the histological activity of the disease and TNFRs up-regulation might modify host response and potentially contribute to liver damage [54].

IL-2 is a cytokine produced by T cells in response to inflammatory stimuli. It induces the surface expression of IL-2 receptor (IL-2R) and, consequently, the production of its soluble form, sIL-2R. The excess of sIL-2R is capable of binding IL-2 and causes the inhibition of an appropriate immune response. IL-2R is the protein that mediates the action of IL-2, which is normally not displayed at a significant number on T and B cell surfaces. Stimulation of the immune system causes two IL-2R changes: more molecules of "IL-2R" expressed on the cell plasma membrane and sIL-2Rα is released by the activated cells into the surrounding fluid [55]. Our results showed that levels of IL-2Rα were elevated in all studied patients with a statistically significant difference in HCC patients when compared to those with PNALT (*p *= 0.001). This could be attributed to the binding of IL-2 due to excess of its receptor and thus inducing an inhibition of the appropriate immune response with subsequent progression of chronic liver disease and the development of HCC. Previous results [13,17,56] are in agreement with ours, where it is was shown that serum levels of sIL-2R are correlated with the histological severity of liver damage in HCV patients, which may be used as a marker in patients at high risk of getting HCC as the highest levels of soluble IL-2R occurred in those patients. The sIL-2R may be an important marker for assessing the phase of active chronic hepatitis and the degree of liver damage [57]. High sIL-2R levels, found in patients with chronic HBV [58,59], were related to the activity of the disease rather than to the virus replication; thus, those levels may be a useful marker of T-cells immune response. In contrast to our results, it was concluded that IL-2R was not detectable in HCC patients in comparison to patients with chronic hepatitis and liver cirrhosis [60]. Regarding the levels of IL-2R in patients with HCC, and in agreement with our findings, there was no statistically significant difference (*p *= 0.62) between its values in men and women [55].

IL-8 is a chemoattractant cytokine which is produced after stimulation with numerous exogenous and endogenous agents. Viruses induce IL-8 production leading to enhanced viral RNA replication and cytopathic effects. Furthermore, evidence was provided that induction of that interleukin was able to attenuate the IFN-α mediated inhibition of viral replication [61]. In the current study, levels of IL-8 were significantly lower in HCC patients than in the other groups (*p *< 0.001). On the contrary, other results found that serum IL-8 levels were markedly elevated in most HCC patients compared with healthy subjects [62] and was found to be over expressed in the HCC tumor cells compared with the non-tumorous livers [63]. Furthermore, multivariate analyses revealed that the levels of the interleukin under consideration may play an important role in the progression and dissemination of HCC and is an independent predictor of long-term survival among those patients. High-serum level of that cytokine may reflect active angiogenesis and rapid tumor growth in HCC. Therefore, targeting IL-8 can represent a potential approach to control angiogenesis and invasion of HCC [62]. In agreement with our results, there was no significant correlation between serum concentration of that cytokine and patient gender (*p *= 0.215) [63].

The present series showed that HCV viral load was significantly correlated with sTNFR-II and IL-8. The production of the latter was found to enhance viral RNA replication [61], thus the low levels of the interleukin in our HCC patients are in accordance with the low HCV viral load. Moreover, there is a good correlation between reduction in virus load and IL-8 level which may indicate that it is related to viral infection rather than to hepatocarcinogenesis.

In the current series, the studied cytokines were significantly correlated to each other. The sFAS was positively correlated with sTNFR-II and IL-2R; sTNFR-II positively correlated with IL-2R and negatively with IL-8; lastly IL-2R and IL-8 were negatively correlated.

Th1 cytokines, which include IL-2R and sTNFR-II, are in favor of an effective immune response against viral infection, whereas Th2 (represented by IL-8 in our study), is in favor of progressive inflammation, continuous cell injury and persistent HCV infection [64].

The depicted correlations could highlight the imbalance between pro- and anti-inflammatory cytokines among patients with CLD and HCC. Furthermore, the rate of progression of CHC to end-stage liver disease might be related to an up-regulation of the TNF-α/Fas pathways [50].

Analysis of sTNFR-II and IL-8 by ROC curves revealed satisfactory values regarding sensitivity and specificity at a cutoff value of ≥ 398 pg/ml and ≤ 290 pg/ml, respectively, when both markers were combined. Therefore, a simultaneous assessment of both sTNFR-II and IL-8 would be beneficial for the diagnosis of HCC; in fact, they were capable of differentiating between patients with PNALT and HCC -- hence, an early detection of HCC among apparently healthy patients with PNALT levels. Nonetheless, these values must be evaluated on a larger scale of patients with various stages of CLD and HCC, in order to be used as new markers for an early detection of HCC.

## Conclusions

Cytokines are involved during disease progression in HCV-infected patients. Early detection of HCC patients is essential in the course of HCV associated CLD and its sequels. IL-2Rα, TNFR-II and sFas were significantly higher, whereas IL-8 values were significantly lower in HCC patients in comparison to the other groups. Our preliminary data revealed that exclusion of HCC among PNALT patients could be predicted when both sTNFR-II and IL-8 are assessed together at a cutoff value ≥ 389 pg/ml and IL-8 < 290 pg/ml, respectively. Nevertheless, further studies with a larger sample size are mandatory to underline the accuracy of our findings before their application at the population level.

## Methods

### Study population

Peripheral blood samples from 79 adult patients with HCV related CLD (with or without HCC) and from 9 healthy subjects (served as the control group) were collected, between April 2005 and June 2006, in the specialized liver clinic of the National Cancer Institute (NCI), Faculty of Medicine, Cairo University, before receiving any treatment. All samples were analyzed for cytokine quantitation. The study was approved by the Investigation and Ethics Committee of the hospital and a written consent was obtained from all the persons involved.

The group size included 30 patients with HCC besides CLD diagnosed by abdominal ultrasonography, triphasic CT abdomen, serum AFP and confirmed histomorphologically; 32 patients with CHC with elevated ALT levels; 15 patients with fibrosis stage ranged from F1-F4; 7 patients with histopathological evidence of cirrhosis (F5-F6); 17 patients patients with PNALT levels for at least 6 months, no organomegaly on ultrasonographic examination and fibrosis stage less than F2, *i.e*., mild fibrosis.

The nine above mentioned healthy subjects (control group) were 50.9 years old (mean) ± 4.6 (standard deviation), with male/female ratio of 7/2, with no clinical or biochemical evidence of liver disease or known medical illness at recruitment and with normal abdominal ultrasonography. All controls were negative for HBV and HCV as evidenced by negative serological markers and negative PCR for HBV and HCV.

Exclusion criteria were: patients with HBV, history of drug hepatotoxicity, autoimmune liver disease and metabolic liver diseases.

### Study design

A detailed history, clinical assessment, biochemical liver profile, abdominal ultrasonography were done to all study groups in addition to serologic testing, virological assay by quantitative PCR (VERSANT HCV RNA 3.0 Assay), HCV genotyping using INNO-LiPA III provided by Innogenetics [65] and histolopathological examination among CLD disease patients to determine the histological activity index (HAI) using the Ishak scoring system [66].

### Cytokine assay

Cytokines were assayed using quantitative ELISA plate method: sIL-2Rα, IL-8, sTNFR-II and sFas using kits provided by Quantikine (R&D Systems, Inc.614 McKinly Place N.E. MN 55413, USA).

### Statistical analysis

The SPSS software package (version 15) was used. Mean ± SD (standard deviation) were computed for the quantitative data. The non-parametric t-test equivalent (Mann-Whitney test) and the non-parametric ANOVA (Kruskal-Wallis test) were used to compare means of, respectively, two or more than two independent groups. Fisher's exact and chi-square tests were used to validate the hypothesis of proportional independency. Correlation analysis was used to detect the association between quantitative data.

## Competing interests

The authors declare that they have no competing interests.

## Authors' contributions

**A-RNZ: **conception and design of the study, drafting the manuscript, revising it critically for important intellectual content. **HMAE-D: **analysis and interpretation of data, drafting the manuscript, revising it critically for important intellectual content, helped in the study supervision. **AAB: **Revision of histological findings of the studied cases, helped in the study supervision. **NAZ: **Provided samples, and collection of data. **WSM: **Participated in the cytokine assaying. **SHE-M: **Participated in the practical part and drafting the manuscript. **SKG: **Participated in the practical part and drafting the manuscript. **GE: **Provided samples, participation in the study design. All authors read and approved the final manuscript.
